# Saccadic and Postsaccadic Disconjugacy in Zebrafish Larvae Suggests Independent Eye Movement Control

**DOI:** 10.3389/fnsys.2016.00080

**Published:** 2016-10-05

**Authors:** Chien-Cheng Chen, Christopher J. Bockisch, Dominik Straumann, Melody Ying-Yu Huang

**Affiliations:** ^1^Department of Neurology, University Hospital Zurich, University of ZurichZurich, Switzerland; ^2^PhD Program in Integrative Molecular Medicine, Life Science Graduate School, University of ZurichZurich, Switzerland; ^3^Department of Ophthalmology, University Hospital Zurich, University of ZurichZurich, Switzerland; ^4^Department of Otorhinolaryngology, University Hospital Zurich, University of ZurichZurich, Switzerland; ^5^Zurich Center for Integrative Human Physiology (ZIHP), University of ZurichZurich, Switzerland; ^6^Neuroscience Center Zurich (ZNZ), University of Zurich and ETH ZurichZurich, Switzerland

**Keywords:** ocular motor, saccades, gaze holding, brainstem, zebrafish, larvae

## Abstract

Spontaneous eye movements of zebrafish larvae in the dark consist of centrifugal saccades that move the eyes from a central to an eccentric position and postsaccadic centripetal drifts. In a previous study, we showed that the fitted single-exponential time constants of the postsaccadic drifts are longer in the temporal-to-nasal (T->N) direction than in the nasal-to-temporal (N->T) direction. In the present study, we further report that saccadic peak velocities are higher and saccadic amplitudes are larger in the N->T direction than in the T->N direction. We investigated the underlying mechanism of this ocular disconjugacy in the dark with a top-down approach. A mathematic ocular motor model, including an eye plant, a set of burst neurons and a velocity-to-position neural integrator (VPNI), was built to simulate the typical larval eye movements in the dark. The modeling parameters, such as VPNI time constants, neural impulse signals generated by the burst neurons and time constants of the eye plant, were iteratively adjusted to fit the average saccadic eye movement. These simulations suggest that four pools of burst neurons and four pools of VPNIs are needed to explain the disconjugate eye movements in our results. A premotor mechanism controls the synchronous timing of binocular saccades, but the pools of burst and integrator neurons in zebrafish larvae seem to be different (and maybe separate) for both eyes and horizontal directions, which leads to the observed ocular disconjugacies during saccades and postsaccadic drifts in the dark.

## Introduction

Many lateral-eyed afoveate animals, such as rabbit (Collewijn, 1969; Baarsma and Collewijn, 1974), rat (Hess et al., 1985; van Alphen et al., 2010), goldfish (Easter, 1971; Beck et al., 2004), and zebrafish (Beck et al., 2004; Huang and Neuhauss, 2008), display yoked eye movements: the two eyes move in the same direction and the timings of binocular saccades are synchronous. Such yoked eye movements help to maintain the spatial relationship between the two visual fields (Voss and Bischof, 2009) as well as to estimate self-motion with respect to the world (Nakayama, 1985; Koenderink, 1986).

Previously it has been shown that, in the dark, 5 days post fertilization (dpf) zebrafish larvae display spontaneous eye movements consisting of centrifugal saccades and subsequent postsaccadic centripetal drifts (see typical eye traces in Figure 1 of Chen et al., 2014). Although the saccade onsets and the directions were consistent in both eyes, the eye drifting in the nasal-to-temporal (N->T) direction moved faster than the eye drifting in the temporal-to-nasal (T->N) direction. Thus, the average time constants of drift (single exponential fits) were 1.8 s in the N->T direction and 3.8 s in the T->N direction (Chen et al., 2014). Since the drifts of the two eyes were disconjugate, it is conceivable that such disconjugacy may also exist in the centrifugal saccades. In the present study, we re-analyzed the data from our previous study (Chen et al., 2014) by calculating saccadic peak velocities and saccadic amplitudes. After confirming both the disconjugate saccadic and postsaccadic eye movements in our results, we raised the following question: what is the underlying mechanism responsible for the yoked but disconjugate eye movements of zebrafish larvae in the dark?

The neuroanatomy of the saccade generation and gaze holding is far better understood in primates than in zebrafish. The actions of four horizontal extraocular muscles (lateral and medial recti muscles of each eye) need to be coordinated to generate yoked horizontal saccadic eye movements (ignoring the smaller contribution of the other extraocular muscles). Excitatory burst neurons in the paramedian pontine reticular formation produce a high frequency discharge, proportional to eye velocity (van Gisbergen et al., 1981), that is sent to the ipsilateral abducens nucleus, where axons contact abducens motor neurons (MN) and internuclear neurons. The internuclear neurons project contralaterally to connect with medial rectus MNs in the oculomotor nucleus (Fuchs et al., 1988). In this way, the excitatory burst produces yoked eye movements by stimulating the ipsilateral lateral rectus and the contralateral medial rectus muscle. To inhibit the antagonistic muscle, inhibitory burst neurons in the rostral medulla project contralaterally to inhibit both MNs and interneurons in the abducens nucleus (Hikosaka et al., 1978; Strassman et al., 1986), thus relaxing both antagonistic muscles. Ocular motoneurons receive a pulse of innervation (velocity command) generated by burst neurons. This causes a phasic contraction of the extraocular muscles so the eyes quickly move to an eccentric eye position. The same pulse signal is also sent to the neural integrator cells in the nucleus prepositus hypoglossi (Cannon and Robinson, 1987; Cheron and Godaux, 1987) and medial vestibular nucleus (McFarland and Fuchs, 1992; McConville et al., 1994) that generate a step of innervation (position command) which further causes a tonic contraction of the extraocular muscles to hold the eye at its new position.

In teleost fish, the regions homologous to the primate saccadic burst generator and neural integrator have also been identified. Stimulation of a small hindbrain region in rhombomere 5 of zebrafish larvae produces binocular, ipsilaterally directed eye movements (Schoonheim et al., 2010), consistent with the action of the saccade burst generator in primates. Likewise, the oculomotor neural integrator for horizontal eye movements has also been identified in goldfish (Pastor et al., 1994; Aksay et al., 2000, 2001).

We addressed our question by simulating spontaneous eye movements in the dark. We adopted a parsimonious ocular motor model composed of three elements (Robinson, 1964): a premotor input, simulating the eye-velocity impulse signal generated by the burst neurons (van Opstal and Goossens, 2008; van der Willigen et al., 2011); a velocity-to-position neural integrator (VPNI) that converts the impulse signal (eye velocity) to a step command (eye position) to keep gaze stable at an eccentric position (Robinson, 1964; Cohen and Komatsuzaki, 1972; Skavenski and Robinson, 1973); and an eye plant model (Keller, 1977; van Gisbergen et al., 1981). The simulated outputs, such as the saccadic peak velocity, the saccadic amplitude and the simulated postsaccadic drift, describe the behavior of eye movements in the dark. These model parameters were iteratively adjusted to fit the average saccadic eye movements of all left eyes in the N->T direction. By analyzing how the model parameters affect the simulated saccadic eye movements, we discuss the origin of the oculomotor disconjugacies in zebrafish larvae.

## Materials and Methods

### Fish Maintenance and Breeding

We re-analyzed the same experimental data as in the study by Chen et al. (2014). Ten 5–6 dpf zebrafish larvae were recorded, but one larva showed no left-to-right saccades. Therefore, only nine fish were studied for this direction.

Larvae were raised according to the protocol described by Mullins et al. (1994). Embryos were placed in E3 medium (5 mM NaCl, 0.17 mM KCl, 0.33 mM CaCl_2_, and 0.33 mM MgSO_4_) with a temperature of 28° and raised under a 10 h dark/ 14 h light cycle (Haffter et al., 1996).

All experiments were performed in accordance with the animal welfare guidelines of the Federal Veterinary Office of Switzerland. Experiments adhered to the Association for Research in Vision and Ophthalmology Statement for the Use of Animals in Ophthalmic and Vision Research.

### Recording of Eye/Body Movements

The eye and body movements of each tested larva were recorded in the dark for 10 min. In order to restrict whole-body motion without constraining eye movements, larva were placed dorsal up in a 21 mm transparent plastic tube filled with 3–3.5% methylcellulose. The plastic tube was placed on a platform where infrared emitting diodes (λ_peak_ = 875 ± 15 nm, OIS-150 880, OSA Opto Light GmbH, Germany) illuminated the larva from below and an infrared sensitive charge-coupled device camera recorded eye and body movements at 40 frames/s from above. A custom-written program in LabVIEW (version 10.0, National Instruments, Austin, TX, USA) extracted the larval eye position in each frame. A binary threshold was used to graphically isolate the eyes along with a user-defined region of interest, with image erosion to smooth the edges. Seen from above, the eyes are oval-shaped, so the eye orientation was determined by calculating the axis with the lowest angular momentum. The eye-position analysis was done on-line and monitored by the experimenter, while body-position analysis was done off-line by calculating the body axis in each frame with a similar image processing algorithm as used for the eye measurement. The larval body movement was used to calculate the eye movement relative to the body as well as to check the timing of body movements (typically short duration vibrations separated by long stationary intervals). All off-line analysis was written in MATLAB (Mathworks, Natick, MA, USA).

### Data Analysis, Saccade Selection and Iterative Fitting Procedure

**Figure 1:**
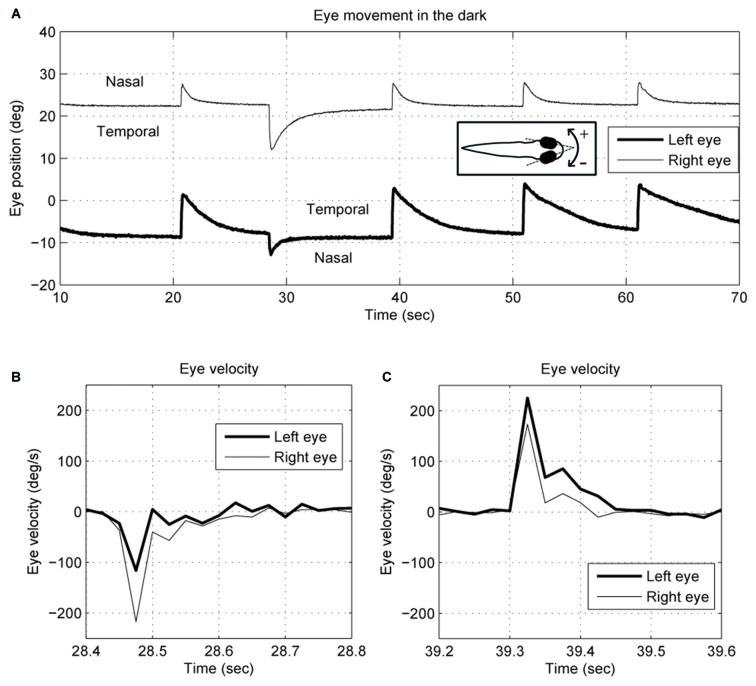
**Typical (A) eye position and (B,C) velocity traces of 5–6-day-old zebrafish larva in the dark**. The two eyes move together in terms of saccade timing and eye-movement direction. However, the saccades and the postsaccadic eye drifts are not conjugate. The right eye trace is depicted with a thin line, and the left eye with a thick line. Note that for the right eye, a nasal movement is to the left, whereas for the left eye a nasal movement is to the right.

We describe eye movements according to whether they are directed nasally or temporally, so, for example, a conjugate movement to the right would be a temporal movement of the right eye and a nasal movement of the left eye. Negative rotations are to the right. Relative to the body, the eyes tend to maintain a steady position when they are rotated nasally. In Figure 1, for example, the right eye drifts back to about 22°, and the left eye about −9°, though these values vary between larvae. Eye-position traces were smoothed using a Gaussian smoothing kernel with a cut-off frequency of 16 Hz. Eye-velocity and acceleration were obtained from the derivatives of eye position. Since the spontaneous eye movements in zebrafish larvae usually start with a saccade to an eccentric position followed by a drift back to a central eye position (Figure 1), the saccade selection was done by: (1) separating the eye position curve into segments based on the eye-movement direction; and (2) identifying a segment as a saccade if the maximum acceleration was >500 deg/s^2^. The other segments would be identified as slow eye drifts. Each eye drift was fitted with a single exponential decay curve to obtain a time constant by using the MATLAB function nlsqnonlin.m.

Saccadic peak velocities were obtained by calculating the maximum absolute velocity of each saccade. The start and end points of saccades were found by determining the first sample when eye velocity reversed direction. Saccadic peak velocity to saccadic amplitude ratios were calculated in each saccade.

### Statistical Analysis

Directional preferences were determined by two tests. In the first test, we separated saccades of each tested larva into two groups based on the N->T and T->N directions. Then, a *t*-test was done between the two groups to check whether there exists a significant difference between the two eye-movement directions.

In the second test, we further checked whether there exists a significant difference between the two eye-movement directions of the two eyes. Thus, we separated saccades of each tested larva into four groups based on the N->T and T->N directions of the two eyes. Then the median value of each group was calculated. A binomial test was used to test the frequency that N->T saccades had a higher peak velocity (or amplitude) than T->N saccades. Since the eye movements of zebrafish larvae are yoked, a T->N movement of one eye co-occurs with an N->T movement of the other eye and vice versa. Thus, the saccadic peak velocity of the T->N movement of the left eye was compared with the one of the N->T movement of the right eye and vice versa.

### Computer Simulation

**Figure 2:**
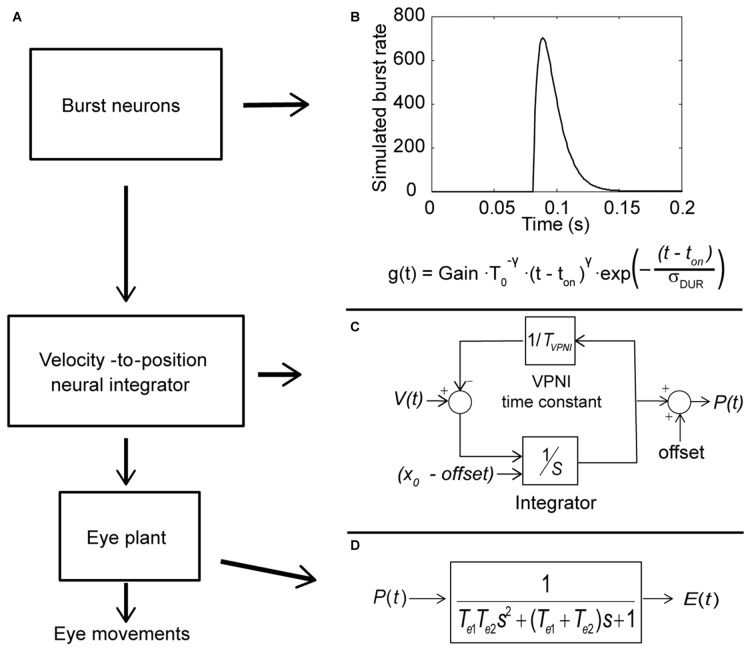
**Conceptual model of the larval ocular motor system for spontaneous eye movements in the dark with Laplace notations. (A)** Model of larval ocular motor system. Burst neurons generate velocity impulse signals to the velocity-to-position neural integrator (VPNI). The VPNI converts the velocity signals to position commands. The eye plant, then, takes the position commands and generates eye movements. **(B)** The firing of burst neurons. The firing is described as a gamma function that can be defined by three parameters: gain, skew and duration (as shown in the equation below), where *t* is time, *t*_on_ is the burst onset, *σ*_DUR_ is the burst duration, the exponent *γ* determines the gamma-burst skewness, and *Gain* determines the amplitude of the gamma function. **(C)** Schematic plot of the VPNI model. The VPNI model receives velocity signals from burst neurons and integrates these signals to position commands. *T*_VPNI_ is the VPNI time constant, V(t) is the velocity signal from burst neurons, *x*_0_ is the initial eye position, offset is the final eye position, and p(t) is the position command. In this study, *x*_0_ and offset are set to zero. **(D)** The mathematical model of the eye plant, described as a second order system that receives position commands p(t) from the VPNI and generates eye movements. The second order system is determined by the two time constants, *T*_e1_ and *T*_e2_.

Computer simulations were done in MATLAB Simulink (Mathworks, Natick, MA, USA). The model includes three subsystems: a set of burst neurons, a VPNI and an eye plant (see Figure 2A). Conceptually, the burst neurons generate eye-velocity impulse signals to quickly change the eye position. Previous studies described such velocity impulse signals as a gamma function (see Figure 2B; van Opstal and Goossens, 2008; van der Willigen et al., 2011):

$$g(t)=Gain\cdot T_{0}^{-\gamma }\cdot {\left(t-t_{\text{on}}\right)}^{\gamma }\cdot \exp\;\left(-\frac{\left(t-t_{\text{on}}\right)}{\sigma_{\text{DUR}}}\right)$$

where *t* is time, *t*_on_ is the burst onset, *σ*_DUR_ is the burst duration, *T*_0_ = *σ*_DUR_ *γ/e* and *t* ≥ *t*_0_. The exponent *γ* determines the gamma-burst skewness, and *Gain* determines the amplitude of the gamma function.

The VPNI converts the eye-velocity impulse signals to eye-position commands. A previous study found that the VPNI in zebrafish larvae is leaky (Miri et al., 2011). Such a leaky VPNI can be modeled by using an integrator with a single time constant as shown in Figure 2C (Chen et al., 2014). These eye-position commands are sent to the eye plant to generate the simulated eye movements. The eye plant in the monkey has been described as a second order system assembled by two time constants *T*_e1_ and *T*_e2_, shown in Figure 2D (Keller, 1977; van Gisbergen et al., 1981). Note that the equivalent studies in fish have not been done, but we assume the results would be similar. *T*_e1_ is approximately equal to the ratio of the viscous drag and the elastic stiffness of the orbital tissues while *T*_e2_ is approximately equal to the ratio of mass of the eye ball and the viscous drag of the orbital tissues. In general, *T*_e1_ is much larger than *T*_e2_ since the mass of the eye is relatively small compared to the effect of the elastic stiffness and the viscous drag of the orbital tissues. For instance, in the model of van Gisbergen et al. (1981) for monkey, *T*_e1_ and *T*_e2_ were set to 0.15 and 0.004, respectively. In this study, we used the product of the two time constants (*T*_e1_ × *T*_e2_), which refers to the ratio of mass of the eye ball and the elastic stiffness of the orbital tissues, and the sum of two time constants (*T*_e1_ + *T*_e2_), which represents the ratio of the viscous drag and the elastic stiffness of the orbital tissues. Since *T*_e1_ is much larger than *T*_e2_, the sum of *T*_e1_ and *T*_e2_ is much larger than the product of *T*_e1_ and *T*_e2_.

## Results

### Spontaneous Eye Movements in the Dark

**Figure 3:**
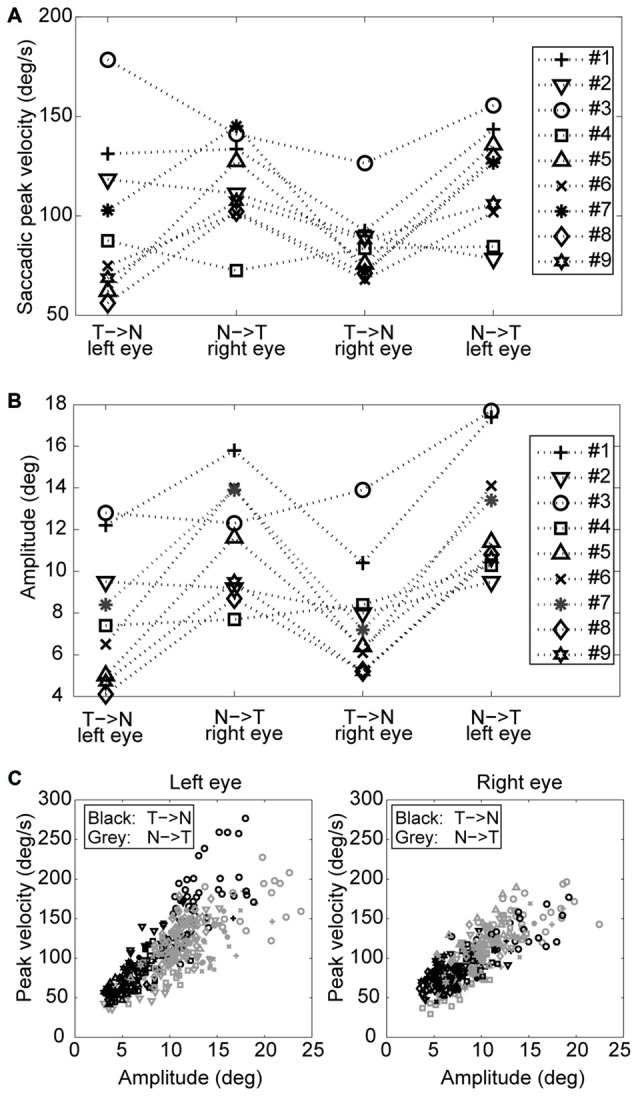
**Saccades of nine tested larvae. (A)** Median saccadic peak velocity, **(B)** amplitude, and **(C)** main sequence of nine tested larvae (two eyes and two directions relative to the head. N->T, nasal to temporal direction; T->N temporal to nasal direction.

An example of the pattern of centrifugal saccades and centripetal drifts is depicted in Figure 1. Clearly, the centrifugal saccades of both eyes were disconjugate. Table 1 lists the median saccadic peak velocity, the median saccadic amplitude and the median ratio of each larva (also see Figure 3). The main sequence is also plotted to visualize the variation between eye-movement directions and larvae (see Figure 3C). Since the medium used to restrain body movements could also affect the eye movement recording, our results can be compared to those by Beck et al. (2004), who restricted only the body movement with agarose. In this measuring method with a higher recording frame rate (60 Hz), the slopes of maximum velocity vs. amplitude (in our case, we use the term “ratio”) in zebrafish larvae were 12–13 (Beck et al., 2004, Figure 5B), which are similar to values in our study (see Table 1, Ratio). Moreover, another study Ma et al. (2014) showed that 5-dpf wild type zebrafish larvae had an average saccadic peak velocity of about 137°/s with a 200 Hz framerate, which is similar to our results. In the same study, the disconjugacy can be found in fast phase velocity during optokinetic stimulation.

**Table 1:** **Saccadic peak velocity, saccadic amplitude and the ratio in eyes and eye-movement directions**.

Subject	Left eye								Right eye							
	Peak velocity		Amplitude		Ratio		Number		Peak velocity		Amplitude		Ratio		Number	
	T-N	N-T	T-N	N-T	T-N	N-T	T-N	N-T	T-N	N-T	T-N	N-T	T-N	N-T	T-N	N-T
1	131.2	143.5	12.2	17.4	10.4	8.4	4	6	92.6	133.6	10.4	15.8	9.5	8.5	6	4
2	118.4	78.5	9.5	9.5	13.2	9.1	23	25	89.6	111.3	8.0	9.2	11.6	12.5	25	23
3	178.5	155.5	12.8	17.7	13.8	9.4	35	20	126.5	141.1	13.9	12.3	9.8	10.9	20	35
4	87.5	84.6	7.4	10.3	11.8	8.6	51	23	83.7	72.5	8.4	7.7	9.7	9.4	23	51
5	62.1	135.8	5.0	11.4	12.4	12.0	23	6	75.9	127.4	6.4	11.6	12.9	11.6	6	23
6	74.9	101.8	6.5	14.1	11.6	8.0	3	17	68.0	101.3	6.1	14.0	11.0	8.3	17	3
7	102.8	126.7	8.4	13.4	12.7	9.1	2	11	72.1	145.1	7.2	13.9	10.5	10.6	11	2
8	56.2	129.3	4.1	10.8	13.4	12.1	10	50	71.1	102.2	5.2	8.7	14.0	12.2	50	10
9	68.7	106.0	4.7	10.6	17.0	10.5	13	9	88.1	107.4	5.2	9.5	16.1	11.1	9	13
Mean	97.8	118.0	7.8	12.8	12.9	9.7	–	–	85.3	115.8	7.9	11.4	11.7	10.6	–	–
STD	39.6	26.6	3.2	3.1	1.8	1.5	–	–	17.8	23.2	2.8	2.8	2.3	1.5	–	–

Using a one-tail *t*-test, we found that the saccadic amplitudes were significantly larger (*p* < 0.05) for saccades in the N->T direction than those in the T->N direction in eight of nine tested larvae, while the saccadic peak velocities were significantly higher (*p* < 0.05) for saccades in the N->T direction than those in the T->N direction in six of nine tested larvae. All non-significant *p-values* were <0.1. Using a binomial test with 18 pairs (since there are two pairs (a T->N movement of one eye co-occurs with a N->T movement of the other eye and vice versa) in one fish, there are 18 pairs in this test), we found that both the median saccadic peak velocities and the median saccadic amplitudes were larger in the N->T direction than those in the T->N direction (for peak velocity, *n* = 18, *Z* = 2.36, *p* = 0.0091; for amplitude, *n* = 18, *Z* = 2.83, *p* = 0.0023).

Postsaccadic exponential centripetal drifts were disconjugate, as we demonstrated previously (Chen et al., 2014; Figure 2D).

### Simulation of Spontaneous Eye Movements in the Dark

**Figure 4:**
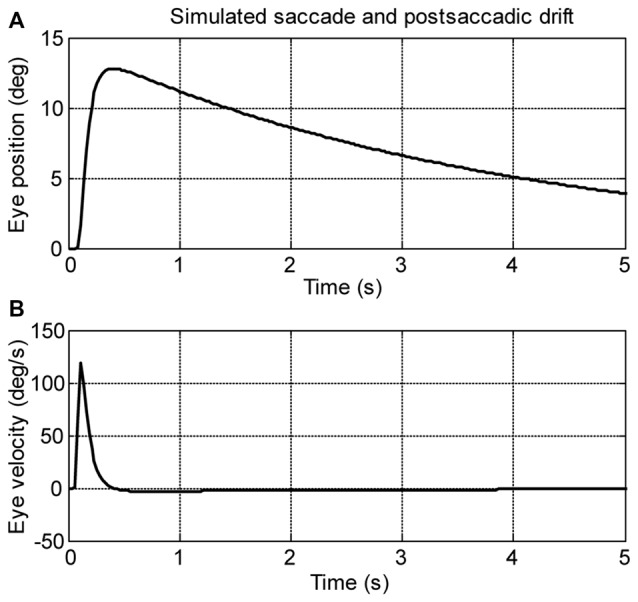
**Simulation saccades of zebrafish larvae in the dark. (A)** Eye position and **(B)** eye velocity of zebrafish larvae in the dark.

A computational model (see Figure 2) was used to simulate larval eye movements in the dark. The model parameters were iteratively adjusted to fit the average median saccade in the N->T direction and the average median eye-drift time constant in the T->N direction of the left eyes. Figure 4 shows the simulated eye movements. The model parameters resulting from the iterative fitting were used for the simulation and are listed and highlighted in gray at the top of Table 2 while the simulated saccade and postsaccadic eye drift in the dark are listed and highlighted in gray at the bottom of Table 2.

**Table 2:** **Model parameter analysis**.

Modeling parameters
Burst	Gain	490	10%					
neurons	Duration	0.01		10%				
(Gamma distribution)	Skew	1.1			10%			
VPNI	*T*_VPNI_	3.8				10%		

Eye plant	*T*_e1_ + *T*_e2_	0.078					10%	
	*T*_e1_*T*_e2_	0.0001						10%
**Simulated eye movements in the dark**
Saccades	Peak velocity	118	10%	7%	3%	0%	−7%	0%
	Amplitude	12.8	10%	10%	4%	1%	−1%	0%

Ratio (Peak velocity/Amplitude)		9.1	0%	−3%	−1%	−1%	−6%	0%

Eye drift	*T*	3.8	0%	0%	0%	10%	0%	0%

*The top of the table indicates the values of the model parameters. The values in gray were obtained by iteratively fitting the empirical data. The bottom of the table shows the simulated outputs and elucidates how each model parameter affects the simulated saccade and postsaccadic drift*.

In order to study how changes of single model parameters affect the simulated outputs, we first increased each parameter by 10% of its original value (see Table 2, top) to record the corresponding changes in the model outputs (see Table 2, bottom).

For the burst neuron related parameters, the simulation shows that a 10% increase in *gain* and *duration* has a direct impact on the saccadic peak velocity and amplitude (change ≥7%), but much less on the saccadic peak velocity to amplitude ratio (change ≤3%). The postsaccadic eye drift time constant (*T*), on the other hand, is barely affected (change <1%). A 10% increase in skew has a low impact on the simulated outputs (change ≤ 4%).

For the VPNI related parameters, a 10% increase in *VPNI time constant* mainly affects the simulated postsaccadic centripetal drift (change = 10%). The effect on the simulated centrifugal saccade is minimal (change ≤1%).

For the eye-plant related parameters, a 10% increase of *T*_e1_ + *T*_e2_ (referring to the ratio of the viscous drag to the elastic stiffness of orbital tissues) lowers the simulated saccadic peak velocity by 7%, the ratio of saccadic peak velocity to amplitude by 6%, but the saccadic amplitude only by 1%. The simulated postsaccadic eye drift is not affected by this change. A 10% increase of *T*_e1_ × *T*_e2_ (referring to the ratio of the mass of the eye ball to the elastic stiffness of orbital tissues) has little or no influence on the simulated outputs (all changes are below 1%).

**Figure 5:**
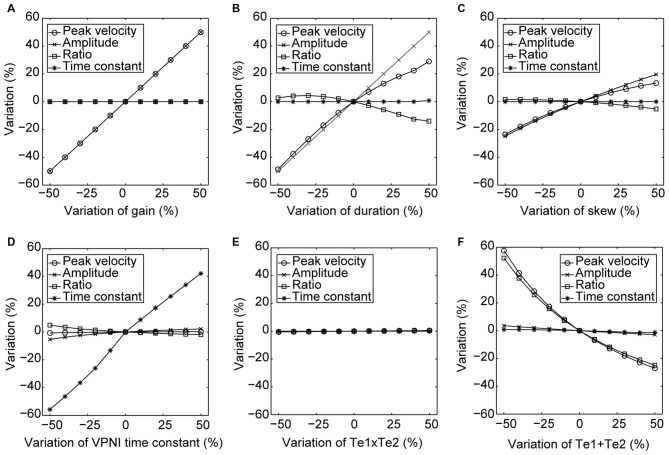
**Changes in the model simulated outputs corresponding to varying model parameters**. The circles depict changes (%) in the saccadic peak velocities, the crosses depict changes (%) in the saccadic amplitudes, the squares depict changes (%) in the ratios of the saccadic peak velocity to the amplitude, and the stars depict changes (%) in the time constants of the postsaccadic eye drifts of the simulated outputs in response to the +50% to −50% changes in **(A)** gain, **(B)** duration, **(C)** skew of the velocity impulse signal generated by the simulated burst neurons, **(D)** VPNI time constant, **(E)** ratio of the velocity drag to the elastic stiffness of the simulated orbital tissues and **(F)** ratio of the mass of the eye ball to the elastic stiffness of the simulated orbital tissues.

Furthermore, to better visualize the dependency of the simulated output on each single model parameter, Figure 5 demonstrates simulated outputs of the model in response to variations of each of the six model parameters within a range from −50% to +50%. The simulation results lead to similar conclusions as indicated in Table 2: simulated saccadic velocities and amplitudes are mainly affected by parameters related to the burst neurons, while the simulated postsaccadic eye drifts are mostly determined by the VPNI time constant.

## Discussion

### Saccadic and Postsaccadic Disconjugacy in Zebrafish Larvae in Dark

In this study, we investigated the disconjugacy of spontaneous horizontal eye movements of zebrafish larvae in the dark. Typical eye movements of larvae in the dark consist of spontaneous centrifugal saccades that move the eyes eccentrically and subsequent centripetal eye drifts that bring the eyes back toward the center (see Figure 1). Although both eyes moved in the same direction and are synchronized, the two eye movements were shown to be disconjugate. The saccadic peak velocities were higher and amplitudes were significantly larger in the (N->T) direction than in the (T->N) direction (Figure 3), while the fitted single-exponential time constants of postsaccadic eye drifts were significantly longer in the T->N direction than in the N->T direction. Such a disconjugate eye movement cannot be due to the mechanical limit of the orbit. In our previous study (Chen et al., 2014), we found that spontaneous saccades in zebrafish larvae occurred mainly in the central area of the eye-movement range. For instance, Figure 4A in our previous study (Chen et al., 2014) is a typical eye movement example in zebrafish larvae. The spontaneous saccades mainly existed in a range of 5–20° of the right eye; the mechanical limit, however, ranges from −5° to 35°. Moreover, as we mentioned in Chen et al. (2014), the VPNI is leaky in zebrafish larvae so that their eyes drift back to a central area after saccades. Thus, to our observation, it is rare that saccades occur in an eccentric area and reach the mechanical limit of the orbit. We also observed a large variation in the saccadic peak velocities among the tested larvae. It could be that the neural and muscular systems of 5–6 dpf larvae have not fully developed. Therefore, some larvae had a strong ocular motor response while others do not.

We further studied the disconjugate eye movements with a top-down approach to identify possible causes for this disconjugacy: a computational ocular motor model composed of burst neurons, a VPNI, and an eye plant was adopted to simulate eye movements in zebrafish larvae in the dark (see Figure 2). After adjusting the model parameters to fit the average N->T centrifugal saccade and the average T->N centripetal postsaccadic eye drift of left larval eyes, we varied each parameter separately to see how changes in single model parameters affect the simulated output. Our results (see Table 2 and Figure 5) showed that the simulated saccadic amplitude is mainly changed by the firing properties of the saccadic burst neurons, which suggests that the observed different saccadic amplitudes in the N->T and T->N directions of the two eyes (see Figure 3) may be attributed to unmatched firings of burst neurons for each eye and for each direction. The simulated saccadic peak velocity, on the other hand, is influenced by both firing properties of burst neurons and the ratio of the viscous drag to the elastic stiffness of the orbital tissues. It is conceivable that the viscosity of the orbital tissues has a direct impact on eye velocities. Thus, an increased ratio of the viscous drag to the elastic stiffness would lower the saccadic peak velocity and therefore change as well the ratio of saccadic peak velocity to the amplitude. In our model, the ratio of the mass of the eye ball to the elastic stiffness of orbital tissues has no influence on the simulated eye movements, which implies that the inertia of the eye should not be responsible for the disconjugate eye movements as its effect is likely too small. The simulated postsaccadic centripetal eye drifts are mainly determined by the VPNI time constant. This suggests that for each eye there exist two distinct neural populations of VPNIs, i.e., a total of four neural populations. Only such a configuration can account for different eye drift time constants in N->T and T->N directions of the two eyes.

### Eye Movement Control

How exactly an ocular motor network controls yoked eye movements has been debated for decades. Two controversial hypotheses of binocular coordination were raised back in the 19th century by the eminent German physiologists, Hermann von Helmholtz and Ewald Hering. While von Helmholtz (1962) argued that the movement control of the two eyes is independent and thus binocular coordination is a learned behavior, Hering (1977) stated that the binocular coordination is an inborn behavior and that two eyes do not move separately but rather the same impulse will direct both eyes to move simultaneously. Subsequently, the latter hypothesis, known as Hering’s Law of Equal Innervation, has generally been favored (Howard and Rogers, 1996); however, both theories have been supported for a variety of reasons and by substantial evidence (for review see King and Zhou, 2000).

**Figure 6:**
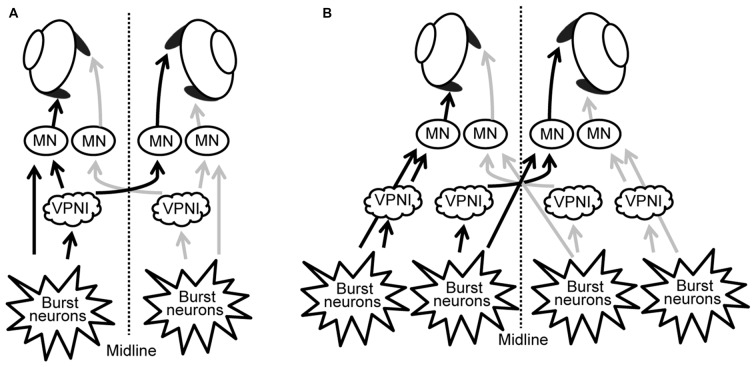
**Scheme of neuronal populations controlling saccades and gaze holding in the brainstem. (A)** Binocular control, where a single command controls the movement of both eyes, similar to Hering’s law in primates. **(B)** Uniocular control, where each eye receives separate movement commands, similar to Helmholtz’s concept. MN, motor neuron; VPNI, velocity-to-position neural integrator.

Our results suggest that, rather than having a single unique control system for both eyes, the two eyes of zebrafish are controlled independently by distinct neuronal populations (see Figures 6A,B). These, in turn, are driven by premotor mechanisms that ensure synchronous timing of binocular saccades in the same horizontal direction.

Evidence in primates has led to a similar conclusion. Pre-motor neurons have been found which preferentially encode the movement or position of one eye (King and Zhou, 2000; Sylvestre et al., 2003; Van Horn and Cullen, 2008; Van Horn et al., 2008; Waitzman et al., 2008). In particular, excitatory burst neurons have been identified which encode monocular saccade velocity, rather than a conjugate command (King and Zhou, 2000), and neurons that are part of the VPNI generally encode the position of only one eye during disjunctive movements (Sylvestre et al., 2003).

The debate between Herring and von Helmholtz concerned primate eye movements, who, being frontal eyed and foveate, have a greater demand for precise synchronization of the movement of both eyes compared to lateral eyed fish. Since the oculomotor system should be able to adapt to changes due to aging and disease, it would be reasonable for eye alignment to be under adaptive control. Recording (Walton and Mustari, 2015) and microstimulation studies (Walton et al., 2013) of the monkey saccade burst generating regions in strabismic monkeys shows that these structures are altered compared to non-strabismic monkeys. These authors suggested that the premotor neurons have binocular connections, though the strength of these connections can be altered, so pools of premotor neurons could be predominately monocular. Our results suggest a similar situation could exist in zebrafish, adding further support for the use of this species as a model for understanding the oculomotor system in primates (Joshua and Lisberger, 2015).

## Author Contributions

DS, C-CC and MY-YH conceived the study. C-CC performed the experiments. C-CC, DS, CJB, and MY-YH analyzed the data. C-CC, DS, CJB, and MY-YH drafted the article. All authors approved the final version of the article.

## Conflict of Interest Statement

The authors declare that the research was conducted in the absence of any commercial or financial relationships that could be construed as a potential conflict of interest.

## Abbreviations

VPNI: Velocity-to-position neural integrator
N->T: Nasal-to-temporal
T->N: Temporal-to nasal.
